# Distinct Patterns of HIV-1 Evolution within Metastatic Tissues in Patients with Non-Hodgkins Lymphoma

**DOI:** 10.1371/journal.pone.0008153

**Published:** 2009-12-03

**Authors:** Marco Salemi, Susanna L. Lamers, Leanne C. Huysentruyt, Derek Galligan, Rebecca R. Gray, Alanna Morris, Michael S. McGrath

**Affiliations:** 1 Department of Pathology Immunology and Laboratory Medicine, University of Florida, Gainesville, Florida, United States of America; 2 BioInfoExperts, Thibodaux, Louisiana, United States of America; 3 Department of Medicine, Hematology and Oncology, University of California San Francisco, San Francisco, California, United States of America; Institute of Infectious Disease and Molecular Medicine, South Africa

## Abstract

Despite highly active antiretroviral therapy (HAART), AIDS related lymphoma (ARL) occurs at a significantly higher rate in patients infected with the Human Immunodeficiency Virus (HIV) than in the general population. HIV-infected macrophages are a known viral reservoir and have been shown to have lymphomagenic potential in SCID mice; therefore, there is an interest in determining if a viral component to lymphomagenesis also exists. We sequenced HIV-1 envelope gp120 clones obtained *post mortem* from several tumor and non-tumor tissues of two patients who died with AIDS-related Non-Hodgkin's lymphoma (ARL-NH). Similar results were found in both patients: 1) high-resolution phylogenetic analysis showed a significant degree of compartmentalization between lymphoma and non-lymphoma viral sub-populations while viral sub-populations from lymph nodes appeared to be intermixed within sequences from tumor and non-tumor tissues, 2) a 100-fold increase in the effective HIV population size in tumor versus non-tumor tissues was associated with the emergence of lymphadenopathy and aggressive metastatic ARL, and 3) HIV gene flow among lymph nodes, normal and metastatic tissues was non-random. The different population dynamics between the viruses found in tumors versus the non-tumor associated viruses suggest that there is a significant relationship between HIV evolution and lymphoma pathogenesis. Moreover, the study indicates that HIV could be used as an effective marker to study the origin and dissemination of lymphomas *in vivo*.

## Introduction

AIDS related lymphoma (ARL) is a disease that occurs in 3–4% of patients infected with the human immunodeficiency virus (HIV) despite the initiation of highly active antiretroviral therapy (HAART) [1]. Since the introduction of HAART, the overall incidence of ARL has been reduced approximately 50%; however, it still occurs at a rate much higher than in non-HIV infected individuals, suggesting a viral component is involved in the development of ARL [1]. The primary difference between lymphoma in non-HIV infected individuals and ARL is that ARLs are uniformly high grade and widely metastatic, with death occurring in as little as two weeks after diagnosis [2]. HIV-related lymphomas are predominately of B-cell origin and often involve extranodal sites, especially the liver and GI tract [3], with about 80% of ARLs arising in the periphery while the remaining occur in the central nervous system (CNS) [4].

It has been proposed that the contribution of HIV to lymphoma pathogenesis may be indirect, related to the duration of immunosuppression, and/or the increased risk for opportunistic infections with oncogenic herpes viruses such as Epstein Barr Virus (EBV) and Human Herpes Virus 8 (HHV-8) [5]. However, several studies show that the expansion of a clonal macrophage population also plays a central initiating role in the early stage of lymphoma development [6]. In non-AIDS settings, patients with follicular lymphoma containing high levels of tumor-associated macrophages (TAM) progress more rapidly than patients with fewer TAM [7], [8], [9], [10].

Zenger et al. [11] isolated TAM from two patients with ARL and implanted them in SCID mice. Interestingly, most of the mice implanted with the human TAM developed aggressive murine lymphomas, which contained the human macrophages within the tumor stroma. PCR analysis confirmed the presence of HIV within the implanted macrophages and revealed that the SCID mouse lymphomas were of murine origin. Additionally, T cells isolated from the same patients with ARL and control macrophages isolated from healthy donors did not induce tumors in the SCID mice, indicating that the HIV positive TAM from the ARLs were responsible for the murine tumor development. Because HIV positive TAM typically express IL-10 and IL-6, cytokines not generally expressed by other forms of HIV infected macrophages (i.e. macrophages in HIV-associated dementia), the finding suggests that HIV indirectly contributes to lymphoma pathogenesis through the creation of tumorigenic microenvironments. In fact, epidemiological studies have shown that increased antigen presentation and macrophage inflammation is significantly protective for lymphoma development, confirming the importance of an anti-inflammatory environment [12].

Molecular studies have identified a subset of large cell lymphomas with prominent *fes*-expressing macrophage populations. *Fes* is a tyrosine protein kinase associated with malignant transformation in animal models, and with intracellular signaling in macrophages initiated by macrophage colony-stimulating factor, GM-CSF and IL-3 [13]. *Ex vivo* studies demonstrated that TAMs from a subset of ARL patients contained monoclonally integrated HIV, upstream to the *c-fes* oncogene, that was associated with polyclonal B-cell lymphoma [14]. In general, the sequential pathogenesis model of ARL suggests the following steps toward disease development: 1) HIV integration near a gene that promotes cellular proliferation; 2) macrophage proliferation; 3) production of lymphostimulatory products in oligoclonal macrophages leading to polyclonal proliferation of B-cells and monoclonal outgrowths; 4) tumorigenesis [3].

Despite an abundance of evidence that implicates HIV-infected macrophages in lymphomagenesis, no studies to date have investigated the evolutionary dynamics of HIV quasispecies infecting tumor and non-tumor tissues from patients with ARL. In the present work, we use high-resolution phylodynamic analysis [15], [16] to track the evolution of HIV-infected tumor and non-tumor tissues harvested at autopsy from two patients who died of lymphoma. The results show that the virus segregates in tumor-associated macrophages and exhibits a distinct population dynamic signature that may be linked to disease onset and progression.

## Results

**Figure 1:**
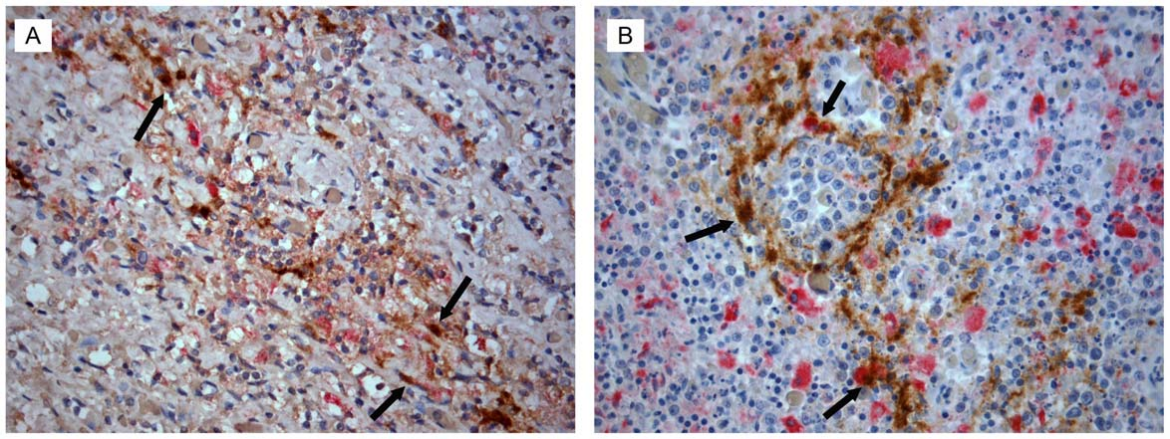
Histopathology of p24 positive macrophages. Tissues from AM and IV were co-stained to identify infiltrating macrophages (CD68, red) and cells productively infected with HIV (p24, brown). Representative tissues of the left axillary lymph node from patient AM (A) and the spleen from patient IV (B) are shown. Examples of double-stained p24 positive macrophages are highlighted by the black arrows. Images are shown at 400X. The distribution and staining pattern similar in all tumor-containing tissues analyzed (not shown).

Multisite autopsy specimens from both patients, AM and IV, were stained with H&E to determine if tumor was present within the collected tissues (Supplemental Table S1). For AM, tumor was identified in the left axillary lymph node, spleen, liver, gastric wall, and diaphragm. For IV, tumor was identified in the right and left axillary lymph nodes, lung, omental, and periaortic lymph node, as well as kidney, spleen, diaphragm, and gastric wall. Tissue sections were also double-stained for the macrophage marker CD68 and the HIV *gag* antigen p24 as described in the Materials and Methods. Our results show that a subset of the TAMs identified with CD68 staining was also positive for HIV p24 (Figure 1). Tissues that were identified as non-involved did not stain positive for p24 and had low levels of CD68 staining (not shown). These data suggest that the macrophages identified with CD68 staining also stain positive for p24 and that in these two patients, HIV-infected macrophages are infiltrating tumor tissues, similar to what we have previously described [11].

### Phylogenetic Analysis of HIV-1 in *Post-Mortem* Tumor and Non-Tumor Tissues

**Figure 2:**
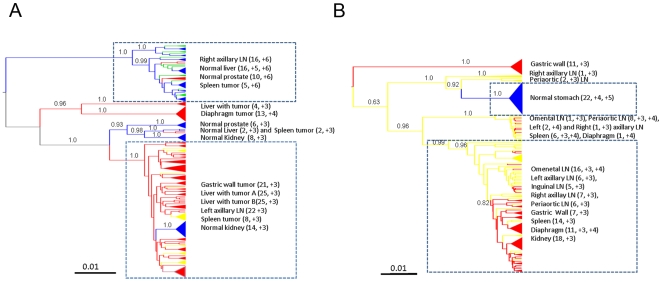
Phylogenetic analysis HIV-1 gp120 sequences from different tissues. Bayesian maximum clade credibility trees assuming a relaxed molecular clock and constant population size coalescent prior generated from the posterior distribution of trees less a 50% burn-in. Branch lengths are shown according to the scale bar at the bottom of each panel, in relative units of time (nucleotide substitutions per site). Posterior probabilities for major nodes are indicated. Internal branches are colored according to the maximum parsimony reconstruction of the ancestral tissue of origin: red = tumor; yellow or green = lymph node; blue = normal tissue. Posterior probability values>80% are shown along the branches. The tissues of origin found within major clades are listed to the right with LN indicating lymph nodes. Number of sequences and V3 loop net charge within the clade for each tissue are given in parentheses. **A.** Subject AM. Yellow and green lineages represent viral strains from right and left axillary lymph nodes, respectively. **B.** Subject IV.

Maximum clade credibility trees, obtained from the posterior distribution of trees, for both subjects are shown in Figure 2. ML and NJ trees, as well as Bayesian trees obtained assuming the non-clock model implemented in MrBayes, gave the same topology (data not shown). HIV genealogies for subjects AM and IV displayed a striking separation of the tumor-associated virus from virus isolated from normal tissues. In subject AM, viral strains from tumor tissues clustered in three distinct and well supported (p>0.95) monophyletic clades. The major tumor clade also contained a sub-clade that joined viral sequences from normal kidney tissue( Figure 2A). Viral sequences from non-tumor tissues formed a well-supported monophyletic clade distinct from the major tumor-associated clades. A few viral strains from spleen tumor also appeared to intermix within the non-tumor tissue clade. Viruses isolated from lymph nodes were interspersed within the largest tumor clade as well as the largest non-tumor tissue clade. Interestingly, viral strains from the left lymph node (where follicular hyperplasia was first diagnosed two months prior to the patient's death) clustered exclusively with the tumor tissue clade, while viruses from the right lymph node clustered exclusively within the non-tumor clade.

HIV sequences from subject IV were obtained from a different set of lymphoid and non-lymphoid tissues than subject AM, but the viral genealogy displayed a remarkably similar compartmentalization between tumor and non-tumor derived strains. The tree showed a monophyletic clade that contained the majority of viral strains from tumor tissues and a distinct lineage containing sequences from normal stomach tissue, all highly supported (p>0.8). Sequences from lymph nodes were interspersed in the tumor clade and viruses from the right and periaortic lymph nodes branched near the non-tumor clade. Sequences from the gastric wall tumor tissue contained two evolutionary distinct and well-supported clades.

In both subjects, prediction of co-receptor usage was consistent with CD68/p24 staining of tumor tissues. All strains belonging to the tumor clade showed low V3-loop charges (≤+4), which are usually associated to macrophage-tropic strains [17], while higher charges (>+4), generally associated with T-cell tropism, were typical of sequences within the major non-tumor clades (Figure 2).

The hypothesis of compartmentalization of the viral quasispecies between tumor and non-tumor tissues was specifically tested using the phylogeny-based test developed by Slatkin and Maddison [18]. Genealogies from both subjects showed a significant lower number of intermixing between HIV-1 sequences from tumor and non-tumor tissues than expected under panmixia (p<0.0001), thus providing strong evidence of viral compartmentalization in tissues with different histopathology. Overall, the results clearly demonstrated that the HIV strains within tumor associated macrophages in both subjects belonged to a unique viral population that was phylogenetically distinct from the strains infecting macrophages and T cells in non-tumor tissues.

### Viral Population Dynamics in Tumor and Non-Tumor Tissues

In order to investigate HIV demographic history in tumor and non-tumor tissues of both subjects, we analyzed separately four sub-datasets, each one including only viral strains clustering in the major tumor or non-tumor clade of each genealogy (clades highlighted by squares in Figure 2). Several coalescent models of viral effective population size (*Ne*) change over time were tested using a Bayesian framework. Bayes Factors (BF) were employed to compare a null model of constant population with parametric (exponential) and non-parametric Bayesian skyline plot (BSP) models of growth. For both subjects, no or weak evidence was found against the null model when viral strains from normal tissues were considered, although the BSP model was slightly above the cutoff for patient AM (Supplemental Table S2). On the other hand, in both subjects the exponential and BSP models fitted the data significantly better than the constant one for the viral sub-population infecting tumor tissues, with the BSP model always displaying the highest marginal likelihood (data not shown). Under the exponential model, Bayesian estimates of HIV effective population size growth rate, as well as 95% high posterior density (95%HPD) intervals of the estimates, were also remarkably similar for both subjects (AM: median 10.2, 95%HPD 6.8–14.4; IV: median 10.7, 95%HPD 6.9–14.9).

**Figure 3:**
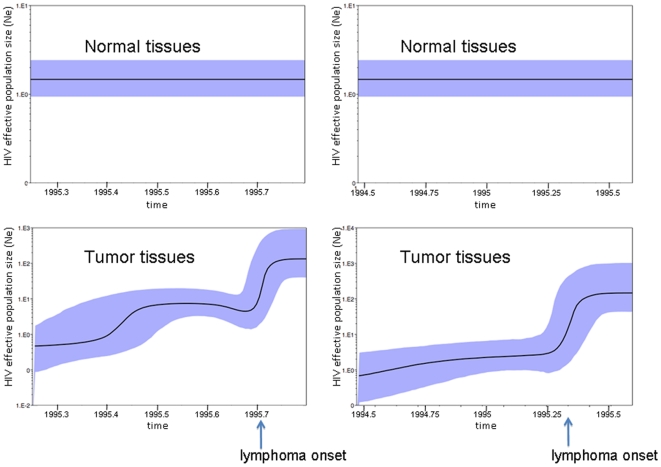
HIV-1 population dynamics in tumor and non-tumor tissues. Bayesian estimates of HIV-1 effective population size (number of infectious genomes effectively contributing to the next generation, y-axis) over time (x-axis) were inferred using the models that fitted best for each data set according to the Bayes Factors test (see Supplemental Table S2). The black line represents the median estimate of the effective population size with the shaded area showing the 95% high posterior density intervals of the estimates. **Top panels.** Constant population size over time of HIV-1 in normal tissues from patient AM (left panel) and IV (right panel). **Bottom panels.** Non-parametric estimates (Bayesian skyline plots) of effective population size change over time for subject AM (left panel) and IV (right panel).

A low and constant *Ne*, estimated between 1–10 effectively infectious genomes, characterized the HIV population dynamic in non-tumor tissues (Figure 3 top panels) during the last year of the patients' life. However, in the tumor tissues, *Ne* increased more than 100-fold during the last three months of the infection coincident to the clinical onset of lymphoma (Figure 3 bottom panels). The 95%HPD intervals of *Ne* for the beginning and ending estimates did not overlap, consistent with the significant BF for the demographic models assuming population growth in these clades.

### Molecular Clock Analysis

One hypothesis that could explain the increase in viral *Ne* within tumor clades is a higher viral evolutionary rate leading to increased diversity and resulting, in turn, into higher estimates of effective population size over time. To address this hypothesis, HIV-1 rate of evolution was investigated by enforcing a local molecular clock on the trees in Figures 2A and 2B for viruses derived from normal and tumor tissues, respectively, and a baseline clock for the remaining part of the tree. The local clock model was then compared with both a strict clock (one evolutionary rate for the whole tree) and a non-clock model. As expected, the non-clock model always fitted the data significantly better than the strict or the local one (Supplemental Table S3). However, the comparison between the strict and local molecular clocks showed that the hypothesis of two different evolutionary rates for the strains replicating in tumor and non-tumor tissues, respectively, did not fit the data significantly better than the strict clock (p>0.05). Therefore, the exponential increase of *Ne* over time in tumor tissues was apparently not related to an increased evolutionary rate of the virus in the infiltrating macrophages.

To investigate further whether the molecular clock was different for synonymous or non-synonymous substitutions, we performed the molecular-clock analysis using only 1st+2^nd^ nonsynonymous codon positions or only synonymous 3^rd^ codon positions (see Methods). In both cases, the strict clock could not be rejected when compared to the local clock model. Moreover, when synonymous 3^rd^ codon positions were analyzed, the strict-clock model did not fit the data significantly worse than the non-clock model (p>0.05). Since the synonymous substitution rate is proportional to the replication rate of the virus [19], the result strongly suggested that in both subjects HIV-1 replicated at a similar rate in tumor and non-tumor tissues.

### Viral Gene Flow among *Post-Mortem* Tissues

**Figure 4:**
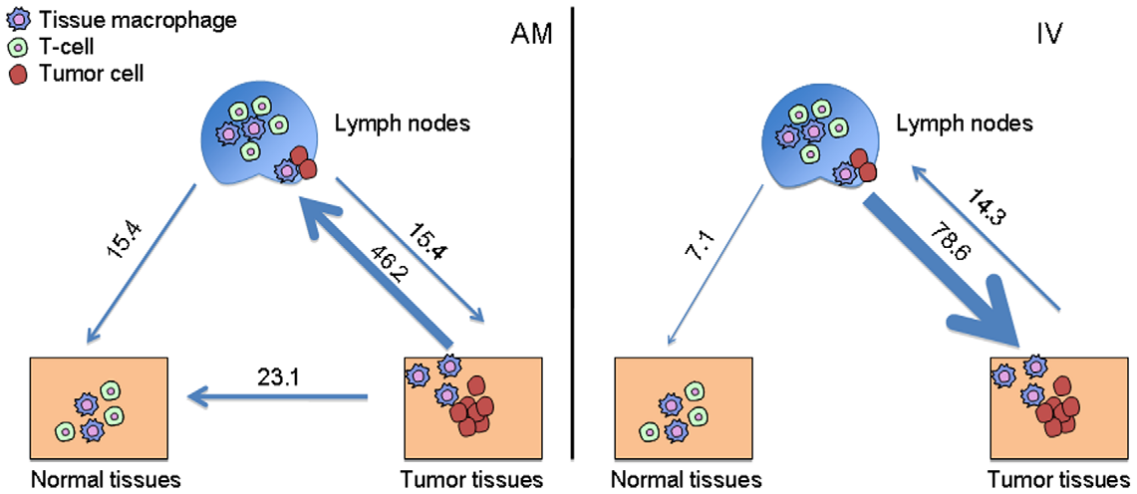
Viral gene flow analysis among different. Light blue arrows represent HIV-1 gene flow from/to normal tissues, tumor tissues and lymph nodes. Viral gene flow among different tissues was inferred with a modified version of the Slatkin and Maddison method^13,28^ from the HIV-1 genealogies including strains from different tissues. Numbers on the arrows represent percentage of observed migration in the genealogies. **Left panel.** Subject AM. **Right panel.** Subject IV.

The HIV-1 genealogies from both subjects showed a high degree of compartmentalization between viral strains from tumor and non-tumor tissues with strains from lymph nodes intermixing between the two (Figure 2). Therefore, we decided to analyze in more detail HIV-1 metapopulation structure by quantifying the relative amount of viral gene flow between tissue pairs. The average number of migration events (viral gene flow) to/from tissues characterized by different histopathology was inferred with a modified version of the Slatkin and Maddison test [16], [18] from the distribution (minus the burnin) of the HIV-1 genealogies obtained by Bayesian inference. The number of migrations was normalized to calculate the percentage of viral genomes flowing between tissues (Figure 4). For both subjects, the majority of gene flow occurred between lymph nodes and the tumor tissues (AM: 61.6%; IV: 92.9%). In subject AM, more viruses were exchanged from the tumor to the lymph nodes (46.9%) than in the opposite direction. In subject IV, the majority of the virus flowed from the lymph nodes to the tumors (78.6%). In both subjects the tumor tissue and the lymph nodes contributed virus to the normal tissue at less than 25% of the total gene flow, gene flow from lymph nodes to normal tissues was generally low (15.4% and 7.1% in AM and IV, respectively), and no flow was observed from normal to tumor tissues. Gene flow counts were all statistically significant when compared with a panmictic model of random flow among tissues (p<0.001). Overall, these patterns echoed the topology of the phylogenetic trees, in which the virus from the tumor and non-tumor tissues appeared highly compartmentalized with viral strains from lymph nodes interspersed within different clades.

## Discussion

The present study is the first to investigate HIV-1 evolutionary patterns among tissues from lymphoma patients and to demonstrate the existence of a distinct viral sub-population at least in part associated with TAMs. Although further work will be required to test the actual lymphomagenic potential of HIV lymphoma-associated quasispecies, our findings have important implications towards a thorough understanding of the nature of tumorigenesis and suggest that HIV-1 DNA could provide a molecular marker capable of tracking the spread of TAMs and thus tumor in vivo. These data may have important implications for studying the role of TAMs in the pathogenesis of cancer metastasis and as targets for macrophage-specific therapeutic development.

The two ARL patients examined died within 2 to 4 weeks of their diagnosis and phylodynamic analysis of HIV sequences from *post mortem* tissues revealed several interesting aspects of viral evolution: 1) both patients displayed remarkably similar patterns suggesting the existence of tumor and non-tumor-specific viral sub-populations, characterized by different growth rates; 2) the results consistently showed that while HIV-1 strains were highly compartmentalized between tumor and non-tumor tissues, strains from lymph nodes were interspersed within the two groups; 3) the presence of two different subpopulations of viruses in the patients with lymphoma is not the result of drug selection since both patients were HAART-naïve, and 4) HIV gene flow among lymphoid and non-lymphoid tissues with different histopathology was non-random. In particular, subject AM showed viral sequences from left and right axillary lymph nodes compartmentalizing, respectively, with tumor and non-tumor tissues.

It is important to notice that although compartmentalization tests clearly showed that the HIV-1 subpopulation infecting tumor tissues significantly differed from the one infecting non-tumor tissues, some intermixing can still be observed. The result is not unexpected. First, occasional exchange of virus (gene-flow) between contiguous tumor and non-tumor cells is possible, as shown by gene-flow analysis (Figure 4). Second, mixed tissue viral branches that occur between the non-tumor and tumor clades may represent a biologically ancestral form of the tumor virus. For example, patient IV showed a population of lymph node, spleen and gastric wall sequences branching out of the tumor clade that likely represented an older population of sequences. Due to the high viral turnover in lymph nodes, a mixture of viral populations from these tissues is expected. In only one case do we see complete tissue compartmentalization, and this is with Patient IV, stomach, non-tumor sequences. Van Marle et al. showed that HIV sequences derived from the gut-associated lymphoid tissues are frequently compartmentalized from PBMC, and even from regions within the gut (stomach, esophagus, duodenum, colon)[20]. In this sense, it is not surprising that normal stomach is compartmentalized from the other tissues in the study, yet it is interesting that nearly half of the tissues from a gastric wall tumor were found within the tumor clade. If gut compartmentalization is common, then the only explanation for sequences in tumor clade to be found in the gastric wall is due to the migration and establishment of a metastatic macrophage-harboring virus from a site of tumorigenesis.

The finding of strongly compartmentalized populations of sequences isolated from different tissues, especially tumor-associated viruses within HAART-naïve ARL patients, suggests two potential biological mechanisms: 1) the virus found in ARL is migrating within metastatic macrophages and/or 2) lymphoma-associated strains of HIV exist. This finding is significant because the identification and characterization of lymphoma-specific viral strains may shed further light into the study of lymphomagenic processes. In particular, such viruses may contain specific protein structures or amino acid substitutions, or may induce expression patterns in macrophages that are associated with lymphoma onset and progression.

One characteristic of end-stage AIDS is the presence of p24 expressing macrophages along with T cell depletion [8], [9]. A recent AIDS autopsy study showed that lymph nodes, spleen and brain contained p24 expressing macrophages[21]. Additionally, previous studies of ARL tissues demonstrated that p24 staining within tumor tissues was predominantly localized to macrophages interspersed in a background of p24-negative lymphocytes. Laser-capture microdissection studies of these tissues localized integrated forms of HIV to TAMs [21], [22]. The histology from patients AM and IV pointed out that a subset of TAMs identified with CD68 staining were also positive for HIV p24, implying that at least some of the HIV sequences generated for this study were derived from tumor associated tissue macrophages. Such conclusion was strengthened as well by the predicted co-receptor usage for the strains amplified from tumor tissues; however, the possibility of harvesting lymphocytes from the tissues collected exists. In one case, viruses from tumor tissues did not map within the tumor clade (5 spleen tumor sequences in patient AM) and contained highly charged V3 loop sequences. It is possible that these represented viruses infecting lymphocytes present in the sample.

Overall, these findings seem to indicate that during lymphoma metastasis, the lymph nodes acted as the conduit of metastatic cells migrating to distinct sites in the body and that the lymph nodes exchanged virus with both non-tumor and tumor associated TAMs. It has been recently proposed that metastasis is initiated by hematopoietic progenitor cells (HPCs) and macrophages that co-migrate to future metastatic sites establish a favorable microenvironment for the seeding of tumor cells [23], [24], [25]. These HPCs and macrophages mobilize in response to growth factors produced by the primary tumor, such as vascular endothelial growth factor A (VEGFA), placental growth factor (PlGF) and transforming growth factor-beta (TGFb), where they establish a pre-metastatic niche. Our data suggest the possibility that a lymphoma specific sub-population specifically infecting TAMs may promote lymphomagenesis. Over time, infected macrophages migrate to future secondary tumor sites where they likely establish a pre-metastatic niche and set up a favorable environment for ARL metastatic cells. Strikingly, we found that the viral effective population size (*Ne*) in the tumor clade for both patients expanded ∼100 fold (from ∼1 to 100) during the course of infection, while the viral population in the non-tumor clade remained constant. Molecular clock analysis revealed that the exponential increase of the *Ne* was not due to an increased evolutionary or replication rate of HIV-1 in tissues with different histopathology. The exponential increase in viral population size, on the other hand, is consistent with an increase number of macrophages within the tumor environment that are susceptible to HIV-1 infection. Human tumors, including lymphomas, secrete chemokines such as monocyte chemotactic protein-1 (MCP-1), macrophage colony stimulating factor (M-CSF), and VEGF, which are believed to be important for the recruitment of macrophages into tumor sites [26], [27]. Therefore, tumor-derived chemokines could be attracting macrophages into the tumor where they would be promoting tumor growth and metastasis through TAM's known pro-tumor properties as well as becoming infected with HIV-1. Further support for this hypothesis is that the majority of the gene flow in both patients was between tumor and lymph nodes, while almost no gene flow was observed between either tumor or lymph nodes and normal tissues. The continuous recruitment-infection of new macrophages in tumor tissues can explain the exponential growth of the tumor-associated viral quasispecies that occurred at the onset of ARL and progressed till patients' death, and suggest that a similar HIV-1 population dynamic in TAMs may underlie ARL onset and progression. One limitation of our study is that only two cases could be included. We believe, however, that the highly significant finding of HIV-1 compartmentalization in tumor and non-tumor tissues in two patients with independent clinical histories and the different numbers and sets of tissues analyzed should strengthen the confidence in our results.

To date, the evolution of HIV infection in macrophages has been primarily addressed in the context of the T-cell-free central nervous system. These studies have identified macrophages as long-lived reservoirs of HIV infection and replication and as sites for increased viral recombination. TAMs are not targeted by HAART. Most importantly, studies have shown that HIV infected macrophages are not prone to cell lysis whereas T-cells are. These facts make TAMs interesting in the context of how virus populations might persist and migrate in the context of macrophage-associated disease processes, such as lymphoma and dementia. The evolution of HIV within TAMs likely represents an entirely different epigenetic event than that found in T-cells and deserves increased attention in future studies.

## Materials and Methods

### Biomaterial

Multi-site frozen autopsy specimens (Supplemental Table S1) of lymphoid and non-lymphoid tissues were obtained through the AIDS and Cancer Specimen Resource (ACSR) (http://acsr.ucsf.edu) from two patients who died with ARL as previously described [21]. Tissues were classified as tumor, non-tumor, or mixed tissues by histological examination, and stained with antibodies to CD68 and HIV p24 as described below. Quantitative HIV genetic studies qualified tissues studied as having >1 copy of HIV/2000 genomic equivalents for further genetic analyses. The ACSR is a National Cancer Institute funded tissue banking program that obtains tissues from patients after appropriate consent and the application of a de-identification procedure before sending the tissues out to ACSR-approved investigators. Clinical histories are handled similarly in a de-identified manner. Patient two-letter designations used throughout this study were generated randomly as shorthand used by technicians who performed the studies so as not to have any correlation to patient information. The ACSR is recognized by the Office of Biorepositories and Biospecimen Research at the National Institutes of Health as being HIPAA compliant and in accordance with the ethical standards of the Declaration of Helsinki. Additionally, all material was obtained under approval from the UCSF committee on human research.

### Subjects

Subject AM died without HAART or cancer chemotherapy. Two months prior to death he presented with lymphadenopathy and peripheral edema with a left axillary lymph node biopsy showing follicular hyperplasia. The subject had a two week history of weight loss and fever with a painful left axillary lymph node and palpable nodes elsewhere. Autopsy findings were significant for EBV negative disseminated large-cell lymphoma involving the stomach, pancreas, spleen, left kidney, all sampled lymph nodes, and portions of the diaphragm, and liver. There was no evidence of tumors in the prostate, right kidney, or brain.

Patient IV died without HAART or cancer chemotherapy. Two weeks prior to death the subject presented with ascites. One week later the subject developed diffuse adenopathy and fevers. High-grade lymphoma was diagnosed in the bone marrow 1 day prior to death. Autopsy findings were significant for high-grade EBV positive lymphoma involving spleen, intestines, kidney, diaphragm, all sampled lymph nodes, and portions of the stomach. The brain showed multinucleated giant cells and perivascular macrophage cuffing consistent with HIV associated encephalitis.

### Histology

Frozen samples from multi-site autopsies were fixed in 10% neutral buffered formalin and embedded in paraffin. The samples were sectioned at 5um, stained with hematoxylin and eosin (H&E), and examined by light microscopy as we previously described ^16^. H&E stained slides were reviewed by a pathologist and classified as non-tumor, tumor, or mixed tissues.

CD68 and p24 immunohistochemistry was performed using fixed samples that were matched to the frozen tissues analyzed by H&E. After deparaffinization, rehydration, washing, and heat-induced epitope retrieval, the slides were treated with mouse anti-HIV p24 (Dako, 1∶10) in 1X wash buffer overnight at 4C. The slides were then rinsed and treated with anti-mouse Envision+HRP, followed by DAB. Slides were then microwaved in antigen retrieval solution to remove the remaining antibodies. After cooling, monoclonal mouse anti-human CD68 clone PGM1 (DAKO, Denmark, 1∶25) in 1X wash buffer was applied for 45 minutes (Dako) at room temperature. Slides were then treated with anti-mouse Envision+AP for 45 minutes followed by Vector Red alkaline phosphatase substrate (Vector Laboratories, Burlingame, CA), counterstained with Mayer's hematoxylin (1∶2) and examined by light microscopy.

### Amplification, Cloning and Sequencing

Amplification, cloning, and sequencing of the envelope gp120 viral protein were analyzed using primers and conditions previously described [21]. Briefly, genomic DNA was extracted from 10–30 mg of the total frozen biopsy tissue from each tissue listed in supplemental table S1 using the QIAmp DNA Mini Kit from Qiagen according to the manufacture's protocol. A 3.3 kb HIV fragment, spanning the env to the 3′LTR region was amplified by PCR. PCR products were cloned into the pCR2.1-TOPO vector. Clones containing the proper insert were identified with PCR. Sequencing was preformed on approximately 20–40 clones derived from each biopsy tissue by MCLab (South San Francisco). Putative recombinant sequences were excluded from further analysis. The final data sets consisted of 197 sequences from subject AM and 146 sequences from subject IV. Multiple-sequence alignments were obtained by codon-alignment with the CLUSTAL algorithm [28], and subsequent manual editing for optimization. Putative recombinant sequences were detected by using the PHI-test based algorithm as previously described [29], [30].

### Phylogenetic Analysis of Non-Recombinant Data Sets

The best fitting nucleotide substitution model was tested with a hierarchical likelihood ratio test, using a neighbor-joining (NJ) tree with LogDet corrected distances [31]. Maximum likelihood (ML) trees were inferred with the selected model and ML-estimated substitution parameters. The heuristic search for the best tree was performed using an NJ tree as a starting tree and the TBR branch-swapping algorithm. Neighbor-Joining (NJ) trees were estimated using pair-wise distances inferred by ML with the best fitting nucleotide substitution model. Calculations were performed with PHYML [32]. Statistical support for internal branches of each tree was obtained by bootstrapping (1000 replicates for the NJ trees; 200 replicates for the ML trees). Each tree was rooted using ML by selecting the rooted tree with the best likelihood under the molecular clock constraint or by outgroup rooting using strains from the other subject. The location of the root was confirmed by inferring rooted Bayesian trees with a relaxed clock model. Since sequences were all collected at the same time point (*post mortem*) we used a strong prior for the evolutionary rate using a previous estimate of 1.2 10^−2^ nucleotide substitutions per site per year based on several independent intra-patient data sets [19], [33]. The Bayesian calculation consisted of 100,000,000 generations Markov Chains Monte Carlo (MCMC) with sampling every 10,000^th^ generation using the BEAST software package version 1.4 [34]. Convergence of the MCMC was assessed by calculating the effective sampling size (ESS) of the combined runs. All parameter estimates showed significant ESS (>300). Bayesian trees were also obtained with the program MrBayes v3.1.2, using the HKY+Γ model running in parallel two MCMC for 10,000,000 generations with sampling every 100^th^ generation [35]. Convergence was assessed by comparing the average standard deviation of split frequencies (*p*<0.0001). Statistical support for each clade in the Bayesian trees was obtained by calculating clade-specific Bayesian posterior probabilities with MrBayes. In each case, ML and Bayesian methods inferred the same topology. For each dataset, the maximum clade credibility tree, which is the tree with the largest product of posterior clade probabilities, was selected from the posterior tree distribution after 10% burn in using the program TreeAnnotator version 1.4.8. Final trees were manipulated in FigTree v.1.1.2 for display (http://evolve.zoo.ox.ac.uk/beast/).

### Coalescent Models

By using coalescent theory, we can infer the demographic history of a population from the genealogical relationships of sampled individuals [36], [37]. A genealogy reconstructed from randomly sampled HIV sequences contains information about population-level processes such as change in population size and growth rate. Given a viral phylogeny *P* and a vector φ representing the parameters of a demographic model *N*(*t*), it is possible to calculate the log of the conditional probability *ln*[φ|*P*]. Bayesian estimates of φ can be found by MCMC sampling procedure. We considered six demographic models for the each data set: constant population size with strict or relaxed molecular clock, exponential growth with strict or relaxed molecular clock, and Bayesian skyline plot (BSP) with strict or relaxed molecular clock. Both parametric (constant or exponential model) and non-parametric (BSP) estimates of demographic history were performed with BEAST version 1.8 by running one MCMC for 100,000,000 generations with sampling every 10,000^th^ generation. Each aligned data set was partitioned in 1^st^+2^nd^ and 3^rd^ codon positions and the parameters of the nucleotide substitution (HKY+Γ+I) and demographic model were estimated independently for ach partition. All parameter estimates showed significant ESS (>300).

While the constant and the exponential model are nested, the BSP is a non-parametric model that cannot be compared with the other two by comparing the mean log posterior probabilities. However, model comparison in a Bayesian framework can be achieved by calculating the Bayes Factor (BF), which is the ratio of the marginal likelihoods with respect to the prior of the two models being compared [38]. We calculated approximate marginal likelihoods for each coalescent model via importance sampling (1000 bootstraps) using the harmonic mean of the sampled likelihoods with the posterior as the importance distribution [39]. The calculations were performed with Tracer 1.4 (http://evolve.zoo.ox.ac.uk/beast/). Twice the difference in *log_e_* space of marginal likelihood between any two models is the Bayes Factor, 2*log_e_*(BF). Evidence against the null model (i.e. the model with lower marginal likelihood) is assessed in the following way: 2>[2*log_e_*(BF)] indicates no evidence against the null model; 10>[2log_e_(BF)]>6 indicates positive to strong evidence against the null model; [2*log_e_*(BF)]>10 indicates very strong evidence against the null model [38].

### Gene Flow Tests and Migration Counts

The hypothesis of compartmentalization, i.e. the existence of distinct HIV-1 sub-populations within different tissues, was tested by the Slatkin and Maddison test [18] for gene flow using the MacClade version 4 program [40]. A one-character data matrix was obtained from the original data set by assigning to each *taxon* in the tree a one-letter code indicating its tissue of origin. The maximum clade credibility tree was obtained from the distribution of phylogenetic trees (minus the burnin) from Bayesian analysis and imported into MacClade. Then, the putative origin of each ancestral sequence (i.e. internal node) in the tree was inferred with the Fitch algorithm by finding the most parsimonious reconstruction (MPR) of the ancestral character. The final result was a tree with colored branches, where each color represented the tissue of origin of the internal node (ancestral sequence) or tip node (actual sequence) subtending that branch. A change in color (i.e. in tissue assignment) between two branches connected through a node represented a migration event from one tissue to another that must have occurred during the genealogical evolution of the sequences under investigation. The tree-length, i.e. the total number of observed migrations in the genealogy, computed by MacClade was then compared to the tree-length distribution of 10,000 trees obtained by random joining-splitting. Observed genealogies significantly shorter than random trees indicated the presence of subdivided populations. Specific migrations among different compartments (states) were traced with the *State changes and stasis* tool (MacClade), which counts the number of changes in a tree for each pair-wise state as previously described [16]. When multiple MPRs were present, the algorithm calculated the average migration count over all possible MPRs for each pair. The resulting pair-wise migration matrix was normalized to obtain the percentage of observed migration to/from different tissues in the tree.

### Molecular Clock Analysis

Three different molecular clock models were tested: 1) a strict molecular clock (SC), which assumes the same evolutionary rate for all lineages, 2) a local molecular clock (LC), which assumes different evolutionary rates for the clade containing HIV-1 sequences isolated from tumor tissues and the one including viral strains isolated from normal tissues and, 3) no clock (NC), which allows for a different evolutionary rate along each branch of the tree. Calculations were performed with the BASEML program of the PAML 4.0 package [41] using the tree topologies estimated with BEAST and MrBayes. The clock hypothesis was investigated at both synonymous and nonsynonymous sites by analyzing separately synonymous 3^rd^ codon positions (3^rd^syn cdp) and 1^st^+2^nd^ codon position (nonsyn cdp) for each alignment. Different clock hypotheses were tested with the likelihood ratio test. Degrees of freedom for the test between global and local clock models were calculated by considering that for a binary tree, a local clock model has *n*-1+*r* free parameters, where *r* is the number of unconstrained relative evolutionary rates [41].

### Prediction of HIV-1 Co-Receptor Usage

Net charge of V3 based on number and position of amino acid residues [K+D]-[D+E] was calculated in HIVbase software [42], and phenotype (co-receptor usage and cell tropism) was predicted according to Briggs et al. algorithm (2000) [17]. The result was confirmed by using the geno2pheno algorithm (http://www.geno2pheno.org/).

## Supporting Information

Table S1 Pathological characteristics of tissues from patient AM and IV. a. Mixed tissues contained both tumor and non-tumor cells within the tissue sample.
(0.04 MB DOC)

Table S2 Bayes Factors comparison of viral growth models in tumor and normal tissues. 1. Null hypothesis: constant viral population size. 2. Alternative hypotheses: exponential growth or Bayesian Skyline Plot (BSP). 3. Bayes factor in loge units. 4. Statistical significance was assessed according to the Bayes factors significance tables in Kass and Raftery (1995).
(0.03 MB DOC)

Table S3 Molecular clock analysis of HIV-1 gp120 intra-host quasispecies. 1. Three molecular clock models were evaluated: a non-clock model (NC), assuming a separate rate for each branch in the tree; a local clock model (LC), assuming two different evolutionary rates for the tumor and non-tumor clade, respectively (see Figure 2), and a strict clock model (SC) assuming one evolutionary rate for the entire tree. 2. *log*_e_(Lk) is the natural logarithm of the likelihood estimated for each model using the trees in Figure 2. 3. Likelihood ratio test (LRT) performed to compare the general (left) vs. the null (right) hypothesis. For each comparison, the best fitting model is the one highlighted in bold.
(0.04 MB DOC)
