# Supplementary material for: Distinct Patterns of HIV-1 Evolution within Metastatic Tissues in Patients with Non-Hodgkins Lymphoma
Source: PLoS One. 2009 Dec 3;4(12):e8153. doi: 10.1371/journal.pone.0008153 (PMC2780293; doi:10.1371/journal.pone.0008153)
Supplement: Table S1 — Pathological characteristics of tissues from patient AM and IV. a. Mixed tissues contained both tumor and non-tumor cells within the tissue sample. (0.04 MB DOC) [file pone.0008153.s001.doc]

**Supplemental Table 1. Pathological characteristics of tissues from patient AM and IV.**

| Patient | Tissue | Presence of tumor |
| --- | --- | --- |
| AM | Left Axillary LN | Mixed^a^ |
|  | Right Axillary LN | inconclusive |
|  | Spleen | yes |
|  | Liver Nodule | yes |
|  | Gastric Wall | yes |
|  | Diaphragm Nodule | yes |
|  | Right kidney | no |
|  | Liver | no |
|  | Diaphragm | no |
|  | Prostate | no |
| IV | Right Axillary LN | mixed |
|  | Left Axillary LN | mixed |
|  | Lung LN | mixed |
|  | Omental LN | mixed |
|  | Periaortic LN | mixed |
|  | Kidney | yes |
|  | Spleen | yes |
|  | Diaphragm | yes |
|  | Gastric Wall | yes |
|  | Stomach | no |
