# Supplementary material for: Distinct Patterns of HIV-1 Evolution within Metastatic Tissues in Patients with Non-Hodgkins Lymphoma
Source: PLoS One. 2009 Dec 3;4(12):e8153. doi: 10.1371/journal.pone.0008153 (PMC2780293; doi:10.1371/journal.pone.0008153)
Supplement: Table S2 — Bayes Factors comparison of viral growth models in tumor and normal tissues. 1. Null hypothesis: constant viral population size. 2. Alternative hypotheses: exponential growth or Bayesian Skyline Plot (BSP). 3. Bayes factor in loge units. 4. Statistical significance was assessed according to the Bayes factors significance tables in Kass and Raftery (1995). (0.03 MB DOC) [file pone.0008153.s002.doc]

**Supplemental Table 2. Bayes Factors comparison of viral growth models in tumor and normal tissues.**

| Patient | Tissues | H0 ^1^ | H1 ^2^ | Bayes Factor ^3^ | Evidence against H0 ^4^ |
| --- | --- | --- | --- | --- | --- |
| AM | Normal | Constant | Exponential | 0.5 | None |
|  | Normal | Constant | BSP | 2.37 | Weakly positive |
|  | Tumor | Constant | Exponential | 13.81 | Very strong |
|  | Tumor | Constant | BSP | 15.42 | Very strong |
| IV | Normal | Constant | Exponential | 0 | None |
|  | Normal | Constant | BSP | 1.24 | Weak |
|  | Tumor | Constant | Exponential | 2.94 | Positive |
|  | Tumor | Constant | BSP | 5.84 | Strong |
