# Supplementary material for: Distinct Patterns of HIV-1 Evolution within Metastatic Tissues in Patients with Non-Hodgkins Lymphoma
Source: PLoS One. 2009 Dec 3;4(12):e8153. doi: 10.1371/journal.pone.0008153 (PMC2780293; doi:10.1371/journal.pone.0008153)
Supplement: Table S3 — Molecular clock analysis of HIV-1 gp120 intra-host quasispecies. 1. Three molecular clock models were evaluated: a non-clock model (NC), assuming a separate rate for each branch in the tree; a local clock model (LC), assuming two different evolutionary rates for the tumor and non-tumor clade, respectively (see Figure 2), and a strict clock model (SC) assuming one evolutionary rate for the entire tree. 2. log e(Lk) is the natural logarithm of the likelihood estimated for each model using the trees in Figure 2. 3. Likelihood ratio test (LRT) performed to compare the general (left) vs. the null (right) hypothesis. For each comparison, the best fitting model is the one highlighted in bold. (0.04 MB DOC) [file pone.0008153.s003.doc]

**Supplemental Table 3. Molecular clock analysis of HIV-1 gp120 intra-host quasispecies.**

| Codon positions (cdp) used | Model ^1^ | *log_e_*(Lk) ^2^ | LRT ^3^ | *P*-value |
| --- | --- | --- | --- | --- |
| Subject AM |  |  |  |  |
| *All* | NC  LC  SC | -5266.5  -5396.2  -5398.0 | **NC** *vs.* SC  **NC** *vs.* LC  LC *vs.* **SC** | 3.1•10^-7^  3.9•10^-7^  0.17 |
| 1^st^ + 2^nd^ cdp | NC  LC  SC | -3195.9  -3304.6  -3305.6 | **NC** *vs.* SC  **NC** *vs.* LC  LC *vs.* **SC** | 8.9•10^-4^  8.5•10^-4^  0.37 |
| Synonymous 3^rd^ cdp | NC  LC  SC | -1610.3  -1690.0  -1690.1 | NC *vs.* **SC**  NC *vs.* **LC**  LC *vs.* **SC** | 0.45  0.41  0.90 |
| Subject IV |  |  |  |  |
| *All* | NC  LC  SC | -5407.3  -5519.0  -5519.1 | **NC** *vs.* SC  **NC** *vs.* LC  LC *vs.* **SC** | 3.0•10^-5^  1.9•10^-5^  0.90 |
| 1^st^ + 2^nd^ cdp | NC  LC  SC | -3361.8  -3468.3  -3468.4 | **NC** *vs.* SC  **NC** *vs.* LC  LC *vs.* **SC** | 2.0•10^-4^  1.3•10^-4^  0.90 |
| Synonymous 3^rd^ cdp | NC  LC  SC | -1586.5  -1671.0  -1673.3 | NC *vs.* **SC**  NC *vs.* **LC**  LC *vs.* **SC** | 0.053  0.068  0.1 |
